# Physical activity for the co-prevention of myopia and obesity in children and adolescents: a scoping review

**DOI:** 10.3389/fpubh.2026.1795516

**Published:** 2026-05-18

**Authors:** Xu Zhang, Sheng Zhou, Meng Zhang

**Affiliations:** 1 Sport Department, Suzhou University of Technology, Suzhou, China; 2 Department of Basic Courses, Suzhou City University, Suzhou, China

**Keywords:** children and adolescents, myopia, obesity, physical activity, outdoor exposure, co-prevention

## Abstract

**Introduction:**

Myopia and obesity have become two of the most prevalent public health challenges among children and adolescents, often co-occurring in the same demographic groups. Traditional prevention strategies typically address these conditions separately, failing to consider their shared lifestyle determinants and potential comorbidity mechanisms. This scoping review aims to examine the role of physical activity and outdoor exposure in the joint prevention of myopia and obesity, synthesizing existing evidence and exploring their associations with comorbidity patterns, potential mechanisms, and integrated prevention frameworks.

**Methods:**

We systematically searched PubMed, Web of Science, and China National Knowledge Infrastructure for observational and interventional studies that concurrently examined physical activity and/or outdoor exposure in relation to myopia- and obesity-related outcomes. We applied a scoping review approach to chart and thematically synthesize the evidence, without conducting a quantitative meta-analysis.

**Results:**

Thirteen studies were included in the analysis. The findings consistently show that myopia–obesity comorbidity is prevalent and increasing among children and adolescents. Insufficient physical activity and reduced outdoor exposure were associated with higher obesity risk and poorer visual outcomes. Longitudinal evidence suggests that outdoor activity not only reduces myopia risk but also modifies the relationship between obesity and myopia. National cohort data indicate that suboptimal school physical activity environments are linked to higher myopia–obesity comorbidity risk. Additionally, cohort studies suggest a bidirectional relationship, with early visual problems predicting subsequent increases in BMI.

**Conclusion:**

Physical activity and outdoor exposure may be key modifiable targets for co-preventing myopia and obesity. The school physical activity environment is particularly significant as a structural determinant in integrated prevention efforts. This review proposes a school-based co-prevention framework that focuses on physical activity as a central behavioral nexus, and further studies are needed to test and strengthen this approach with longitudinal and intervention research.

## 1 Introduction

Myopia and obesity have emerged as two of the fastest-growing and most burdensome chronic health challenges among children and adolescents worldwide (1), characterized by evident trends toward widespread prevalence and an earlier age of onset (2, 3). Childhood obesity substantially increases the risk of adulthood cardiovascular disease (4), type 2 diabetes (5), and metabolic syndrome, and is also closely associated with poorer mental health, reduced academic performance, and impaired social functioning (6). Concurrently, myopia has become one of the most prevalent visual health problems among young populations. The early onset of high myopia markedly elevates the lifetime risk of retinal detachment, myopic maculopathy, and irreversible visual impairment (7). In several regions, particularly in East Asia, the dual burden of childhood obesity and myopia has posed a significant challenge to educational systems, public health infrastructures, and long-term socioeconomic development.

While childhood myopia and obesity differ substantially in their clinical manifestations and proximate pathophysiology, they exhibit highly overlapping behavioral and environmental risk profiles. Existing research (8) consistently identifies increased sedentary behavior and screen time, reduced physical activity and outdoor exposure, and heightened academic pressure as important correlates or contributing factors associated with excessive weight gain and myopia development. Within the context of modern lifestyles, prolonged screen use and restricted opportunities for physical activity place children in a dual state of low energy expenditure and insufficient outdoor exposure, which amplifies the risks of abnormal weight trajectories and impaired visual development. These patterns suggest that childhood obesity and myopia may not be entirely independent health problems, but instead may be linked through partially shared modifiable determinants that could be addressed using integrated strategies. However, current prevention and control practices primarily approach childhood obesity and myopia as separate public health issues. Obesity prevention efforts largely focus on dietary interventions and physical activity promotion, whereas myopia control strategies typically emphasize visual behavior management, eye health education, and clinical or optical interventions. Such single-outcome–oriented approaches often fail to account for the systemic nature of children's daily behavior patterns and the environmental constraints that shape them. This segregated approach limits the synergistic potential and long-term sustainability of public health interventions in real-world settings. Given the increasing aggregation of multiple health risks in childhood, there is a growing need to transition from disease-specific prevention toward integrated health promotion frameworks. In this review, co-prevention refers to integrated prevention strategies that aim to address myopia and obesity simultaneously through shared modifiable determinants.

Physical activity and outdoor exposure are widely regarded as key modifiable factors that simultaneously influence weight regulation and visual development in children. Existing evidence has established the central role of physical activity in improving energy metabolism, limiting excess adiposity, and preventing childhood obesity. Concurrently, research over the past decade has consistently shown that increased time spent outside significantly reduces the risk of myopia onset and slows myopia progression. Physical activity and outdoor exposure not only contribute to obesity prevention by increasing energy expenditure, reducing sedentary behavior, and improving metabolic profiles, but also contribute to visual development and myopia prevention by enhancing natural light exposure, reducing sustained near-work demands, and improving accommodative function. Consequently, physical activity and outdoor exposure have been proposed as potential behavioral factors relevant to the joint prevention of childhood obesity and myopia.

Despite this growing body of evidence, existing studies have developed along two parallel yet relatively independent lines of research—“physical activity–obesity” and “outdoor activity–myopia.” Investigations jointly examining myopia- and obesity-related outcomes from a comorbidity or integrated prevention perspective remain limited. Emerging evidence (9) suggests that overweight status may increase the risk of incident myopia and that outdoor activity can substantially modify this adverse association (10). Conversely, early visual problems may also be associated with subsequent weight trajectories by restricting participation in physical activity and reshaping daily behavior patterns. These findings thus indicate that the co-occurrence of myopia and obesity may not be simply coincidental but rather involves bidirectional pathways and complex interactions. However, the overall evidence landscape, study design characteristics, exposure measurements, and key mechanistic pathways related to these associations have not yet been systematically mapped, hindering the development of integrative intervention frameworks.

Children's physical activity and outdoor exposure are highly dependent on their home and educational environments, particularly structural factors such as school physical education curricula, recess and activity policies, and the availability of outdoor spaces and sports facilities. A small number of studies have recently begun to examine school physical activity environments in relation to myopia–obesity comorbidity, suggesting that upstream institutional and environmental determinants may be associated with children's daily activity patterns and may represent an important contextual factor in integrated prevention efforts. However, this emerging body of evidence has not yet been synthesized within a unified conceptual framework. Accordingly, this scoping review aimed to systematically synthesize evidence on physical activity, outdoor exposure, and school physical activity environments in relation to myopia and obesity among children and adolescents. The objective was to chart the evidence landscape of the field and identify potential mechanisms and integrated prevention frameworks.

## 2 Methods

### 2.1 Study design and methodological framework

This study adopted a scoping review methodology to systematically map and synthesize evidence on physical activity-related research in the field of myopia–obesity co-prevention among children and adolescents. A scoping review approach was selected not only because of the substantial heterogeneity in study designs, exposure measurements, and outcome indicators, but also because the relevant evidence remains fragmented across two traditionally separate lines of research, namely physical activity–obesity and outdoor activity–myopia. In this context, a scoping review is particularly suitable because it allows evidence on both conditions to be identified, mapped, and integrated within a common co-prevention perspective, while also accommodating studies on shared exposures, comorbidity patterns, and pathway-related mechanisms. This approach is therefore appropriate for characterizing the breadth of the existing research, clarifying the structure of available evidence, and identifying key themes and knowledge gaps, rather than simply quantitatively synthesizing intervention effects.

The review followed the methodological framework originally proposed by Arksey and O'Malley (11), and incorporated the enhancements suggested by Levac et al. and the Joanna Briggs Institute (JBI) guidelines for scoping reviews. The core stages involved identifying the research questions, systematically searching the literature, selecting eligible studies, charting the data, and collating, summarizing, and synthesizing the evidence. The reporting of this scoping review followed the Preferred Reporting Items for Systematic Reviews and Meta-Analyses extension for Scoping Reviews (PRISMA-ScR) guideline (12). To enhance transparency in study identification and selection, the screening process was presented using an adapted PRISMA 2020 flow diagram. A completed PRISMA-ScR checklist is provided in the Supplementary Materials.

This scoping review was registered on the Open Science Framework (OSF; registration doi: 10.17605/OSF.IO/3SC5J) and is publicly available at:https://osf.io/hy6xj?view_only=4a0f716458064d05b7fe45e7ce266e0d.

#### 2.1.1 Conceptual framework

To systematically characterize the relevance of physical activity to the relationship between myopia and obesity among children and adolescents, we developed a three-tier evidence framework to guide study selection, evidence extraction, and thematic synthesis. This framework delineated the scope of the review across the following three interrelated levels:

1) Comorbidity-oriented evidence—studies concurrently measuring myopia- and obesity-related outcomes within the same population and explicitly examining their co-occurrence, comorbidity, or interrelationships.
2) Shared-exposure evidence—studies examining physical activity, outdoor exposure, or school physical activity environments as primary exposures and analyzing their associations with myopia-related and obesity-related outcomes (even if comorbidity was not explicitly defined). These studies were considered to provide cross-cutting evidence relevant to integrated prevention.
3) Mechanistic and pathway-oriented evidence—studies investigating effect modification, bidirectional associations, or pathway-related relationships involving school environments, behavioral patterns, and health outcomes.

This tiered framework emphasizes that the relationship between myopia and obesity cannot be adequately understood by simply juxtaposing two disease-specific evidence bases, but requires the identification of overlapping exposure structures, interactive mechanisms, and upstream environmental determinants. This framework guided the thematic classification and systematic synthesis of the included studies.

#### 2.1.2 Research questions

This scoping review was guided by the overarching aim of examining the role of physical activity in the co-prevention of myopia and obesity among children and adolescents. From the perspectives of evidence structure, association patterns, and integrated prevention, the review addressed the following research questions:

1) Evidence landscape and study characteristics

What is the current landscape of research examining physical activity, outdoor exposure, and school physical activity environments in relation to myopia and obesity among children and adolescents?

2) Comorbidity and shared-exposure evidence base

Do existing studies indicate the co-occurrence or comorbidity of myopia and overweight/obesity among children and adolescents? To what extent have studies incorporated physical activity and outdoor exposure into analytical frameworks examining myopia- and obesity-related outcomes, thereby providing a shared-exposure evidence base for integrated prevention?

3) Physical activity as a cross-cutting behavioral nexus

Is there evidence of effect modification or bidirectional associations suggesting that physical activity may have an important cross-cutting role in the co-prevention context of myopia and obesity?

4) School context and co-prevention–oriented evidence integration

Have existing studies examined school physical activity environments or physical activity policies as structural factors associated with myopia, obesity, or their comorbidity? Does current evidence enable the identification of key elements and major gaps in supporting integrated prevention to inform the development of a school-based, physical activity-centered co-prevention framework?

### 2.2 Eligibility criteria

Eligibility criteria were defined using the Population–Concept–Context (PCC) framework (13). Explicit inclusion and exclusion criteria were defined for the study population, movement-related exposure concept, outcome scope, and study design. Studies were eligible if they involved children or adolescents, examined physical activity, outdoor exposure, or other movement-related factors in relation to myopia- and obesity-related outcomes, and provided evidence relevant to co-occurrence, shared exposures, pathway-related associations, or integrated prevention. We excluded studies focusing exclusively on adults, studies assessing only anthropometric indicators in relation to myopia without any movement-related factor, studies reporting only one outcome domain, and non-original publications.

#### 2.2.1 Studies

Studies were eligible if they included children and adolescents aged 18 years or younger, regardless of sex, geographic region, or ethnicity. Studies exclusively involving adults or older populations were excluded.

#### 2.2.2 Concept

Eligible studies were required to examine at least one modifiable exposure related to physical activity, including but not limited to: overall physical activity levels, moderate-to-vigorous physical activity, participation in sports, exercise, or physical education classes, recess or school-based physical activity, outdoor activity time or outdoor light exposure, and school physical activity environments or the provision of physical activity opportunities.

Studies were excluded if they focused solely on associations between anthropometric indicators [e.g., body mass index (BMI) or other body shape indices] and myopia without incorporating any physical activity- or outdoor-related exposures.

#### 2.2.3 Context

This review focused on studies that addressed myopia- and obesity-related outcomes within the same investigation, or that provided cross-cutting evidence relevant to integrated prevention from the perspectives of shared exposures or pathway-related evidence.

Myopia-related outcomes included, but were not limited to, myopia incidence or prevalence, spherical equivalent refraction, uncorrected or corrected visual acuity, axial length, and other ocular biometric parameters.

Obesity-related outcomes included, but were not limited to, classification of overweight/obesity status, body mass index, body weight, waist circumference, and related anthropometric indicators.

In accordance with the tiered evidence framework described above, studies were eligible for inclusion if they met at least one of the following criteria:

1) explicitly examined myopia–obesity comorbidity or simultaneously analyzed both outcomes;
2) assessed physical activity–, outdoor exposure-, or movement-related factors in relation to myopia- and obesity-related outcomes within the same study; or
3) investigated effect modification, bidirectional associations, or pathway-oriented mechanisms involving school physical activity environments and related behavioral factors.

Studies were excluded if they met any of the following criteria:

1) included only adult populations;
2) examined only anthropometric indicators in relation to myopia without assessing any physical activity-, outdoor exposure-, or other movement-related factor;
3) reported only myopia-related outcomes or only obesity-related outcomes without relevance to co-occurrence, shared exposures, or integrated prevention; or
4) were non-original publications, including reviews, meta-analyses, conference abstracts, animal studies, or articles without accessible full texts.

#### 2.2.4 Study designs

Original empirical studies were eligible for inclusion, including randomized controlled trials, quasi-experimental studies, cohort studies, case-control studies, and cross-sectional studies. Reviews, meta-analyses, conference abstracts, animal studies, and articles without accessible full texts were excluded. In keeping with the scoping review design, no formal risk-of-bias or methodological quality appraisal was conducted. The purpose of this review was to map, categorize, and synthesize the available evidence rather than to estimate pooled effects or determine the certainty of intervention effectiveness.

### 2.3 Literature screening and data extraction

We systematically searched PubMed, Web of Science Core Collection, and China National Knowledge Infrastructure (CNKI) from database inception to January 1, 2026. The search strategy combined controlled vocabulary terms (e.g., Medical Subject Headings, where applicable) and free-text keywords related to children and adolescents, physical activity/exercise/outdoor activity, and myopia- and obesity-related outcomes. The full database-specific search strategies, including keyword blocks, Boolean operators, and final search strings, are provided in the Supplementary Materials (Supplemental Table 1). Language restrictions were limited to English and Chinese, and only original research articles were considered eligible for screening.

Two reviewers (XZ and MZ) independently screened the titles, abstracts, full texts, and supplementary materials of all retrieved articles and extracted relevant data. Any disagreements were resolved through discussion with a third reviewer (SZ). Formal inter-rater reliability statistics were not calculated. The reference lists of all eligible articles were manually searched to identify additional potentially relevant studies, and supplementary searches were also conducted on the official websites of relevant journals.

All records were imported into EndNote X9.1 for management and duplicate removal. Studies were selected based on the predefined inclusion and exclusion criteria by sequentially screening titles, abstracts, and full texts. Extracted data were entered into Microsoft Excel spreadsheets and summarized descriptively.

The following information was extracted from each eligible study: first author, year of publication, study design, country, age range, sample size, parameters of myopia and weight-related outcomes, characteristics of the exercise intervention (type, frequency, intensity, and duration), control conditions, and primary outcome measures.

To enhance the consistency and comparability of intervention data, a standardized approach was applied during the data extraction process. We systematically recorded the key intervention characteristics for each study, including intensity, duration, and adherence. The intensity of the intervention was categorized into low, moderate, or high based on the frequency and intensity of physical activity (e.g., high intensity was defined as at least 60 min of vigorous exercise per day). Duration was reported as the total time spent on the intervention per day or week (e.g., 30–60 min per day). Adherence was measured as the percentage of participants who completed the recommended intervention dose (e.g., 80% adherence means 80% of participants met the prescribed physical activity amount). This standardized method of data extraction allows for clearer cross-study comparisons and reduces the variability in reporting intervention characteristics.

## 3 Results

### 3.1 Study selection

The database search identified 9,289 records, including 5,894 from PubMed, 2,653 from the Web of Science Core Collection, and 742 from the China National Knowledge Infrastructure. After merging all records and removing duplicates, 6,696 unique articles were retained for title and abstract screening. Of these, 6,535 records were excluded during the initial screening. Full texts of the remaining 161 articles were retrieved and assessed for eligibility; all were available in full.

During title and abstract screening, the literature scope was further refined to focus on the role of physical activity in the joint prevention of myopia and obesity among children and adolescents. Specifically, studies reporting only myopia-related or only obesity-related outcomes were excluded, as were studies that did not consider modifiable lifestyle factors (e.g., physical activity, exercise, outdoor activities, or school physical activity environments) as primary research components. Articles that clearly did not fulfill the target age range or research topic criteria were also excluded at this stage. Following this process, 161 articles were retained for full-text eligibility assessment.

**Figure 1:**
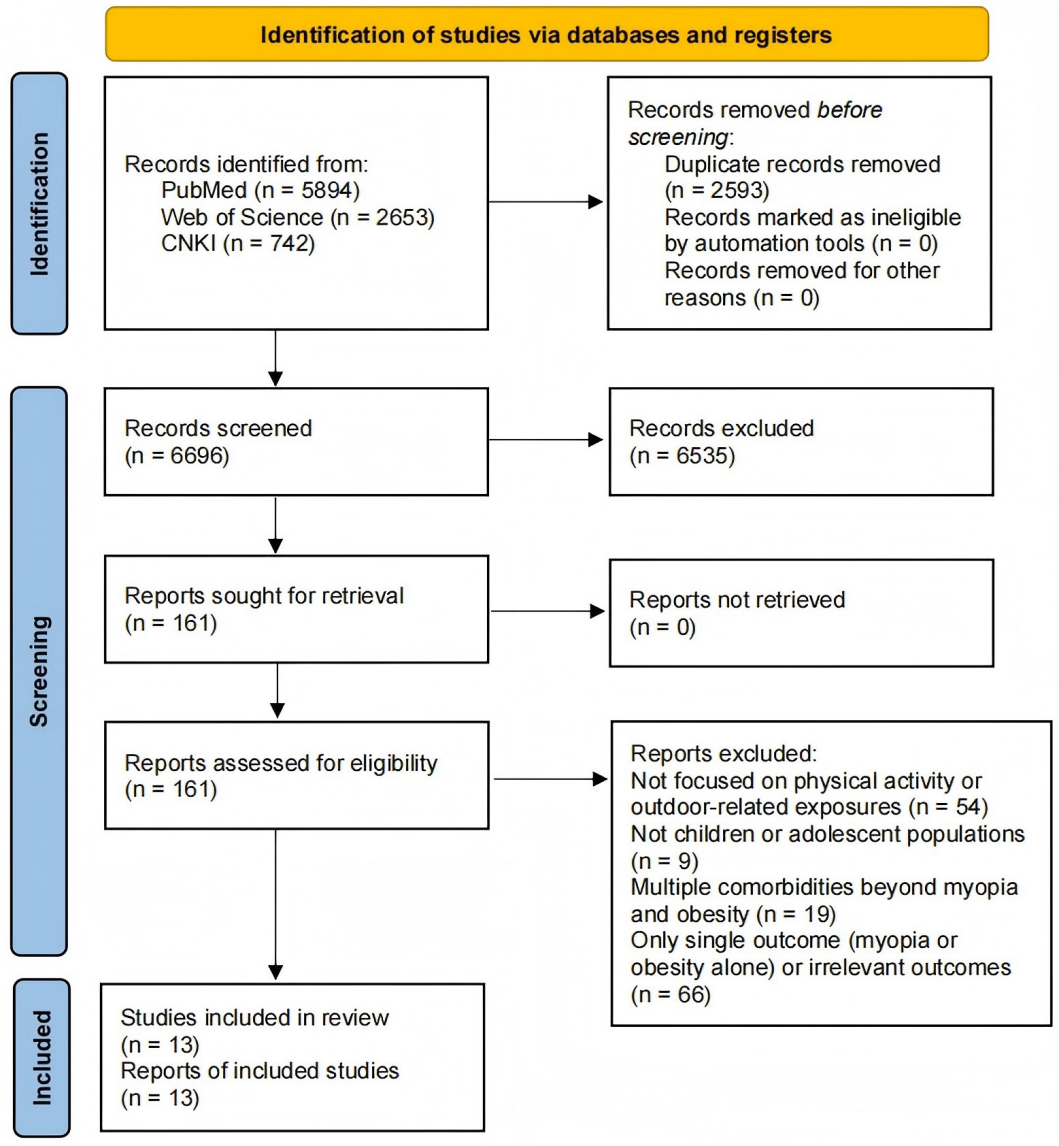
Study selection flow diagram adapted from the PRISMA 2020 template.

After full-text review, 146 studies were excluded for failing to meet the inclusion criteria. The final sample for the scoping review included 13 studies. The detailed study selection process and the number of records at each stage are presented in Figure 1.

### 3.2 Characteristics of the included studies

All 13 included studies focused on physical activity-related factors among children and adolescents and simultaneously examined myopia- and obesity-related outcomes. The characteristics of the included studies, including key exposure and intervention features as well as representative quantitative findings, are summarized in Table 1.

**Table 1:** Characteristics of studies included in the scoping review.

No.	References	Country/ region	Population	Age range (years)	Sample size (n)	Study design	Myopia-relatedmeasures	Obesity-relatedmeasures	Comorbiditydefined	Physical activity/ outdoor exposure/ intervention	Main objective	Key findings (including representative quantitative results)
1	Yang et al. (10)	China (Shanghai)	Schoolchildren (non-myopic at baseline)	6–9	4,683	Prospective cohort study (1–year follow–up)	Spherical equivalent refraction; axial length	BMI	No	Time spent outdoors (min/day)	To examine whether outdoor time moderates the association between early overweight and incident myopia.	Greater outdoor time was associated with lower myopia risk. Outdoor time significantly moderated the overweight–myopia association, with a relative risk (RR) reduction to 0.61 (95% CI 0.38–0.99) for children spending >120 min outdoors. A 70.5% reduction in myopia onset was observed.
2	Liu S. et al. (26)	China (Sichuan)	Children and adolescents	6–18	33,158	Repeated cross–sectional study	Poor vision prevalence	BMI	No	Daytime outdoor activity; moderate–to–vigorous physical activity	To compare pre– and post–pandemic trends in overweight/obesity and poor vision and associated factors.	Outdoor activity and exercise were important correlates of myopia and obesity.Outdoor activity time (≥ 3 h/day) is associated with reduced risk of overweight/obesity (OR = 1.18, *P* < 0.05). This was also found to reduce the risk of poor vision, with higher outdoor activity associated with improved visual outcomes.
3	Dang et al. (27)	China (9 provinces)	Children and adolescents	6–18	9,814/ 5,077	National follow–up study	Visual acuity; non–cycloplegic refraction; spherical equivalent	BMI	Yes	School physical activity environment indicators	To assess prevalence and progression of comorbid myopia and obesity and their associations with school PA environment.	Unfavorable school physical activity environments significantly increased the risk of comorbid myopia and obesity (OR = 1.85, 95% CI: 1.42–2.42), with similar associations observed in the incidence of obesity (OR = 1.86, 95% CI: 1.26–2.75) and myopia progression (OR = 1.29, 95% CI: 1.01–1.65).
4	Drews–Botsch C & Harrington S (28)	Ireland	Children (Growing Up in Ireland cohort)	3–9	6,621	Longitudinal cohort (secondary analysis)	Caregiver–reported early vision problems	BMI; incident overweight/ obesity	No	Physical activity and sedentary behavior	To examine whether early vision problems increase later obesity risk and the role of PA.	Among children with a BMI under the 85th percentile at age 5, those with early vision problems had a 40% higher chance of becoming overweight or obese by age 9 (adjusted OR = 1.39, 95% CI: 1.04–1.85) compared to those without early vision issues
5	Yin et al. (29)	China (nationwide)	Children and adolescents	6–17	35,108	Nationwide cross–sectional study	Myopia prevalence; spherical equivalent	BMI; body fat percentage	No	Moderate–to–vigorous physical activity	To evaluate the association between weight status and myopia.	In girls aged 6–8, those who were overweight or obese had 1.33 times higher odds of developing myopia (OR = 1.33, 95% CI: 1.01–1.76) and 1.71 times higher odds of developing mild myopia (OR = 1.71, 95% CI: 1.14–2.55) compared to their normal–weight peers. Similarly, in the 9–11 age group, overweight or obese girls had 1.22 times higher odds of myopia (OR = 1.22, 95% CI: 1.03–1.44) and 1.31 times higher odds of mild myopia (OR = 1.31, 95% CI: 1.06–1.62) compared to those with normal weight
6	Zong et al. (30)	China (Guangdong)	Preschool children	3–6	23,992	Cross–sectional study	Not directly measured	BMI	No	Outdoor activity time	To explore associations between outdoor activity and health outcomes.	The study shows that increased screen time is linked to a higher risk of being overweight (OR = 1.21, 95% CI: 1.09–1.34), while more outdoor activity is associated with better physical health (β = 0.06, *P* < 0.001).
7	Zhang (31)	China (Inner Mongolia)	Primary and secondary school students	7–18	139,630	Cross–sectional study	Visual acuity; non–cycloplegic refraction	BMI	Yes	Daily ≥60 min moderate–to–vigorous physical activity	To describe comorbidity of myopia and obesity and lifestyle modifiers.	In 2021, the co–morbidity rate of myopia and obesity in Inner Mongolia was 13.7%, with boys having a higher rate (15.5%) than girls (11.8%). Unhealthy lifestyles (OR = 1.24, 95% CI: 1.19–1.29) and moderate lifestyles (OR = 1.15, 95% CI: 1.10–1.19) were linked to higher co–morbidity rates.
8	Dong (32)	China (Xi'an)	Students	9–18	5,768	Cross–sectional study	Visual acuity; spherical equivalent	BMI	Yes	Outdoor activity; MVPA	To explore the effects of screen and outdoor time on comorbidity.	In 2019–2021, the co–morbidity rate of screening myopia and overweight/obesity in Xi'an was 25.3%. Video time ≥2 h/day was a risk factor (OR = 2.111), while outdoor activity time ≥2 h/day was a protective factor (OR = 0.531) for primary school children.
9	Zheng et al. (33)	China	Schoolchildren	7–18	3,658	Cross–sectional pilot study	Spherical equivalent; axial length	BMI	No	Outdoor and study time (covariates)	To examine nonlinear associations between BMI and myopia.	The study found an inverse L–shaped association between BMI and myopia. For participants with BMI < 25 kg/m^2^, each 1 kg/m^2^ increase in BMI increased the risk of myopia by 24.4% (OR = 1.244, 95% CI: 1.211–1.277, p < 0.001). However, no significant association was found for those with BMI ≥25 kg/m^2^
10	Wang et al. (34)	China (Beijing)	Schoolchildren	6–14	Planned ≥4,500	Cluster randomized controlled trial protocol	Visual acuity; refraction	BMI; waist circumference; body composition	Yes	2 h outdoor activity + 1 h physical activity daily	To develop and test an integrated intervention for comorbidity.	Protocol only.
11	Yang (35)	China (Beijing)	Schoolchildren	6–13	1,406	Randomized controlled trial (1 year)	Visual acuity; screening myopia	BMI	Yes	≥2 h outdoor physical activity daily	To evaluate effects of an integrated intervention.	In the intervention group, BMI, waist circumference, hip circumference, and body fat percentage all decreased after the intervention, but none of these changes were statistically significant (t/Z values for BMI = −0.03, waist = −0.36, hip = −0.30, body fat percentage = −0.01, *P*–values > 0.05).
12	Zhang (36)	China	Children and adolescents	6–18	N/A	Indicator development study	Incident myopia; spherical equivalent	BMI; waist–to–height ratio	Yes	Outdoor activity ≥2 h/day	To develop RE–AIM–based evaluation indicators.	Established implementation outcome indicators.
13	Drury et al. (37)	Singapore	Children and parents	5–7	≈60	Community–based pilot intervention + qualitative study	Not assessed	Not assessed	No	Structured outdoor community activities	To assess feasibility and acceptability of outdoor interventions.	Structured outdoor activities were feasible and well accepted.

BMI, body mass index; SE, spherical equivalent; MVPA, moderate-to-vigorous physical activity; PA, physical activity; OR, odds ratio; RR, relative risk; CI, confidence interval.

Participants ranged in age from 3 to 18 years, including preschool children, primary school students, and adolescents in middle and high school. Most studies were conducted in China, with additional studies from Ireland, Singapore, and the United States. Sample sizes varied substantially, ranging from several hundred participants in school- or community-based studies to tens of thousands of children and adolescents derived from national surveillance and longitudinal monitoring systems.

Regarding study design, the included studies were predominantly observational. Designs encompassed prospective cohort studies, repeated cross-sectional studies, national follow-up studies, secondary analyses of surveillance system data, and multiple cross-sectional investigations. We also identified a limited number of intervention-related studies, including one randomized controlled trial, one cluster randomized trial protocol using a hybrid effectiveness-implementation design, and one community-based pilot intervention combined with qualitative focus group research. Overall, the current evidence base remains largely observational, with relatively few intervention studies.

Myopia-related outcome measures included prevalence assessed by vision screening or refractive examination, spherical equivalent refraction, and axial length. Obesity-related indicators primarily included BMI and overweight/obesity classification, with some studies also assessing waist circumference, body fat percentage, and related anthropometric measures. All studies assessed at least one myopia-related and one obesity-related indicator within the same analytical framework.

All studies incorporated at least one physical activity-related variable, including moderate-to-vigorous physical activity, participation in physical education classes, daily exercise duration, outdoor activity time, and school physical activity environment indicators. Most studies considered multiple lifestyle factors, including physical activity, outdoor exposure, sedentary behavior, and screen time, and typically evaluated physical activity within integrated behavioral or environmental models.

### 3.3 Epidemiological characteristics of myopia–obesity comorbidity and the role of physical activity

Several of the studies documented the co-occurrence of myopia and overweight/obesity among children and adolescents across various regions and age groups. A national follow-up study in China reported a marked increase in the prevalence of comorbid myopia and overweight/obesity, from 11.1% in 2019 to 17.9% in 2020, with an incidence of 10.9% during the same period. The most pronounced increase was observed among children aged 6–8 years, suggesting early school age may be a critical window for the emergence of comorbidity.

Regional and repeated cross-sectional studies consistently demonstrated a relatively high coexistence of myopia and overweight/obesity in school populations. Approximately 13%−27% of children with myopia were concurrently overweight or obese across different samples. Furthermore, non-linear patterns were observed across educational stages, and the independent prevalence of obesity and poor vision exhibited parallel temporal trends, indicating potential shared determinants. One large nationwide cross-sectional study revealed complex, non-linear associations between BMI and myopia, including an inverted L-shaped relationship, suggesting that the strength and direction of the association may vary across different BMI ranges.

Most studies examined physical activity or outdoor exposure as lifestyle factors associated with myopia- and obesity-related outcomes. Overall, lower levels of physical activity, less outdoor exposure, and longer durations spent sedentary or using screens were consistently associated with increased risks of overweight/obesity and poor visual outcomes.

Among preschool children, increased outdoor activity time was positively associated with general physical health indicators, whereas greater screen exposure was associated with an increased risk of overweight. Among school-aged children and adolescents, higher levels of moderate-to-vigorous physical activity, more frequent participation in physical education classes, and longer daily outdoor time were associated with reduced risks of overweight/obesity and were important correlates of myopia and visual impairment. Multiple cross-sectional studies reported that, after adjusting for age, sex, and selected lifestyle and socioeconomic factors, children with higher physical activity levels or longer outdoor exposure had lower myopia prevalence and more favorable anthropometric profiles, including lower BMI, body fat percentage, and waist circumference.

Evidence also indicated heterogeneity in the obesity–myopia association. Collectively, these findings indicate that physical activity and outdoor exposure are consistently linked to components of the comorbidity, although the direction and magnitude of obesity–myopia associations may vary depending on population characteristics and measurement approaches.

### 3.4 Environmental, moderating, and intervention-related evidence on myopia–obesity comorbidity

Longitudinal evidence highlighted the moderating role of outdoor activity in the association between overweight and myopia. In a prospective cohort of children without myopia at baseline, overweight status was associated with a higher risk of incident myopia, whereas this association was attenuated among children with longer outdoor exposure. Additive interaction analyses suggested that approximately 70.5% of the combined effect could be attributed to the interaction contribution of outdoor activity. This pattern may reflect the combined influence of greater light exposure, reduced near-work demands, and healthier movement-related behavior patterns among children with more outdoor time.

From a structural perspective, the school physical activity environment emerged as an important contextual correlate of myopia–obesity comorbidity. A national follow-up study developed a composite indicator that encompassed the frequency and intensity of physical education classes, recess activity, the duration of school-based physical activity, and the organization of sports activities. In this context, a suboptimal school physical activity environment referred to less favorable provision across these components, including limited physical education opportunities, insufficient recess activity, shorter school-based physical activity time, and weaker organization of sports activities. Such an environment was significantly associated with a greater risk of comorbid myopia and overweight/obesity, and was also linked to increased risks of overweight/obesity and myopia progression. These associations remained robust following adjustment for individual-level physical activity and family-related factors, highlighting the potential relevance of supportive school environments to the joint prevention of myopia and obesity.

Intervention or implementation-focused research regarding myopia–obesity comorbidity was limited. One cluster randomized controlled trial protocol described the development of a school-based intervention that integrated extended outdoor exposure with structured physical activity to prevent myopia and obesity. A randomized controlled intervention study in Beijing evaluated the effects of a one-year comprehensive program that promoted at least 2 h of outdoor physical activity daily. The results showed non-significant reductions in BMI and adiposity indicators in the intervention group, and a general decline in visual acuity was observed in intervention and control groups.

Beyond efficacy-focused interventions, the included research also addressed implementation and feasibility. One study developed indicators for evaluating integrated myopia–obesity interventions based on the RE-AIM (Reach, Effectiveness, Adoption, Implementation, and Maintenance) framework, providing methodological support for future implementation research. Additionally, a Singaporean community-based pilot study explored the feasibility and acceptability of structured outdoor programs for children and parents, identifying facilitators such as family engagement and organized activities, as well as barriers, including academic commitments. Together, these studies highlight that intervention research in this field is in its nascent stages and highlight the need for rigorously designed, large-scale trials and implementation-oriented studies.

## 4 Discussion

### 4.1 Overall interpretation of the main findings and evidence structure

This scoping review suggests that research on the co-prevention of myopia and obesity in children and adolescents is still at an early stage. Of the 13 included studies, 10 were observational, whereas only 3 were intervention- or implementation-related. Importantly, among these 3 studies, only 1 was a completed randomized controlled trial, while the others were a trial protocol and a community-based pilot/feasibility study. This pattern indicates that the current evidence base is still dominated by descriptive and association-based research, limiting causal inference and the translation of findings into scalable practice. Although the available evidence consistently identifies physical activity and outdoor exposure as promising shared targets for both myopia and obesity, substantial gaps remain in longitudinal validation, integrated intervention development, and school-based implementation studies that simultaneously assess both outcomes.

Rather than describing a vague “sensitivity” to lifestyle change, the available evidence more specifically suggests that myopia and overweight/obesity are consistently linked to modifiable behavioral and environmental exposures. Across the included studies, longitudinal, cross-sectional, and school-based evidence converged in showing that outdoor activity, physical activity patterns, and supportive school activity environments were relevant to comorbidity risk and related outcomes. For example, one study found that greater outdoor time was associated with reduced myopia incidence (OR = 0.79, 95% CI: 0.63–0.99), while another demonstrated that increased physical activity was linked to a significant reduction in overweight/obesity (OR = 1.22, 95% CI: 1.01–1.44). Furthermore, studies from various regions, including China, Singapore, and Ireland, emphasized the role of structured outdoor activities, with one indicating that structured school physical activity programs could reduce comorbidity risk by 31% (OR = 0.69, 95% CI: 0.47–0.98). Thus, the co-occurrence of myopia and obesity appears not only epidemiologically observable, but also meaningfully connected to modifiable daily behaviors and contextual conditions. These patterns reinforce the rationale for integrated prevention, while the detailed quantitative findings are presented in the Results section.

While research on physical activity for obesity prevention and outdoor exposure for myopia is well-established, studies focusing on joint prevention are still limited. Few studies explicitly assess both myopia and obesity outcomes together, and there is a lack of intervention or implementation research in this area. As such, the current literature offers a starting point for integrated prevention but lacks the comprehensive evidence needed to support myopia–obesity co-prevention.

Most included studies assessed physical activity alongside other lifestyle factors, such as sedentary behavior, screen time, and sleep, reflecting the integrated nature of children's health behaviors. However, few studies have used time-use models to examine how reallocating time across these behaviors influences both myopia and obesity. Future research should incorporate these models to clarify the behavioral trade-offs and better understand how daily activity structures impact both outcomes.

A major limitation of the current evidence is the significant measurement heterogeneity across studies. Physical activity and outdoor exposure were assessed using various methods, such as self-reports, activity frequency, and school environment indicators, with few studies using objective or device-based measures. Similarly, myopia and obesity outcomes varied, with myopia assessed through caregiver reports, vision screenings, and refractive measures, while obesity was measured using BMI, waist circumference, and other indicators. This variability hampers comparability and complicates the identification of consistent exposure–outcome patterns. The main measurement approaches are summarized in Supplementary Table 3.

### 4.2 Physical activity as a behavioral nexus for the joint prevention of myopia and obesity

**Figure 2:**
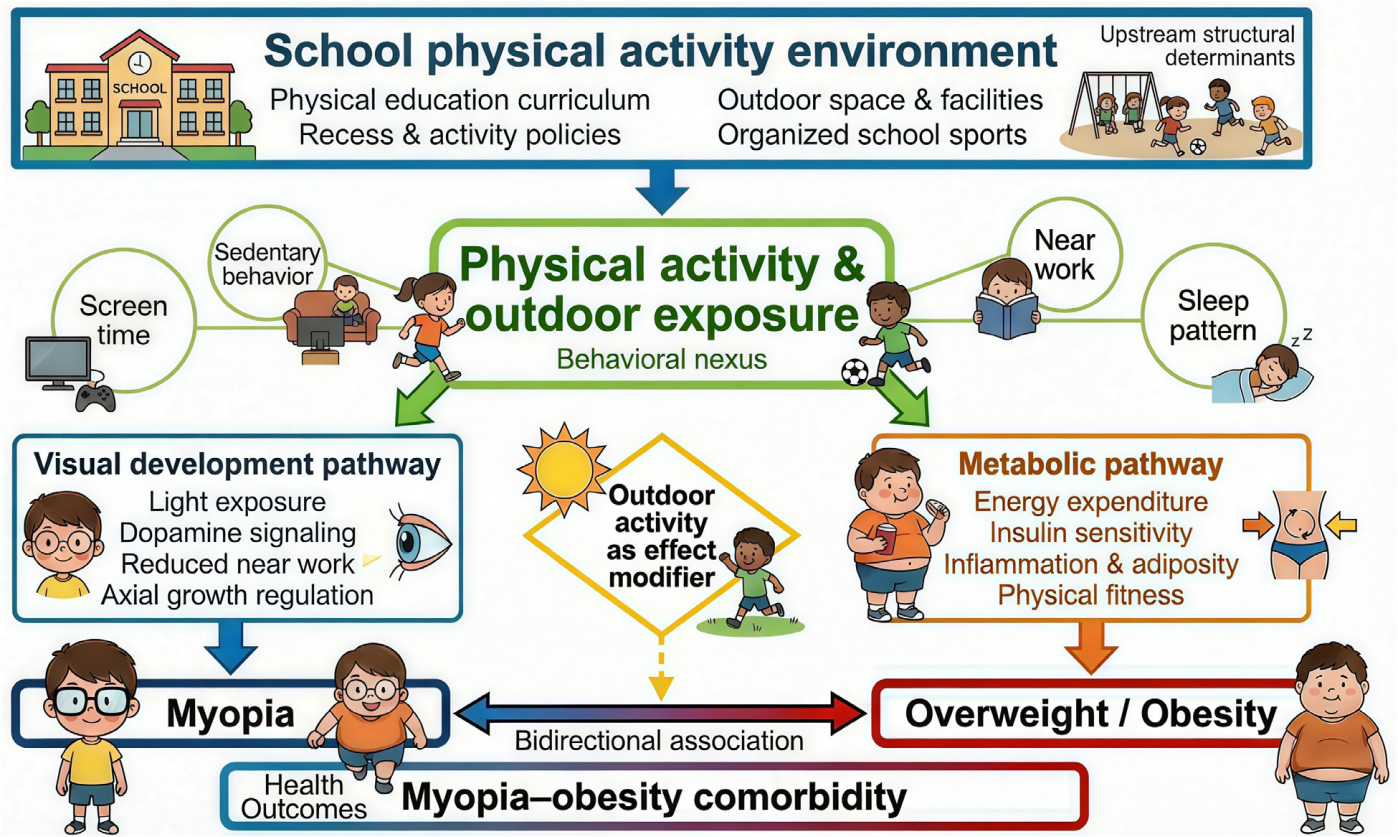
A school-based co-prevention framework situating physical activity within the myopia–obesity context. The figure illustrates the “behavioral nexus” through which physical activity and outdoor exposure interact with sedentary behavior, screen time, and near-work patterns to influence both visual and metabolic health outcomes.

The studies included in this review suggest that physical activity may function as a behavioral nexus at the intersection of visual and metabolic health. In this review, behavioral nexus refers to a shared movement-related behavioral system comprising physical activity, outdoor exposure, sedentary behavior, screen time, and near-work allocation, through which daily behavior patterns may simultaneously influence myopia-related and obesity-related outcomes. Operationally, this nexus links visual health through greater light exposure and reduced sustained near-work demands, while linking metabolic health through increased energy expenditure, healthier body composition, and improved metabolic regulation. This concept is further illustrated in the proposed school-based co-prevention framework (Figure 2), in which physical activity and outdoor exposure are positioned as central behavioral nodes connecting environmental conditions with dual health outcomes.

BMI served as the primary proxy for adiposity in most included studies; however, the reported associations between BMI and myopia were inconsistent. For example, a study of 983 children aged 5–18 years identified an association between elevated BMI and high myopia (14). Conversely, research among Korean adult men suggested that individuals with lower BMI and a tall, slender body type might be more susceptible to myopia, potentially due to longer axial length (15). Another study of 3,915 US. adolescents aged 12–19 years reported a positive association between the body roundness index and myopia among those with higher levels of physical activity (16). Collectively, these findings suggest that the link between isolated anthropometric indicators (such as BMI) and myopia is unlikely to be linear or unidirectional, and may be strongly influenced by age, behavioral characteristics, and lifestyle patterns. These inconsistencies also suggest that BMI alone may be too limited to capture the broader developmental and behavioral pathways potentially linking adiposity to visual health.

Within this broader behavioral-developmental framework, a relatively high or low BMI may reflect distinct physical activity profiles and lifestyle structures. Higher BMI is often associated with insufficient physical activity and increased sedentary behavior, whereas lower BMI or a tall and slender body type may also coincide with limited sports participation, reduced outdoor exposure, and prolonged near-work activities. From this perspective, physical activity and the broader daily behavioral system in which it is embedded may serve as key mediators or moderators that shape the association between myopia and obesity. Physical activity serves a dual role, determining energy balance and body composition trajectories on the one hand, and simultaneously modulating visual load, outdoor light exposure, and time allocated to either sedentary or movement-related behaviors on the other, potentially establishing behavioral links between adiposity and refractive development.

These observations suggest that myopia and overweight/obesity in children and adolescents should not be viewed as two separate conditions that merely co-occur, but rather as clustered outcomes shaped by overlapping behavioral and environmental determinants. From a public health perspective, this distinction supports a shift from parallel single-outcome prevention toward integrated prevention strategies that can address shared risk structures within the same child, school, and family contexts. Such an approach may improve implementation efficiency and generate broader health gains under the same resource investment. The existing evidence (17, 18) consistently indicates that physical activity and outdoor exposure are among the most overlapping and actionable factors influencing myopia and obesity, providing a strong rationale for positioning them as central entry points for joint prevention.

### 4.3 Mechanisms linking physical activity to myopia and obesity

Existing evidence has established the biological and behavioral mechanisms by which physical activity influences obesity and myopia. In terms of obesity prevention, physical activity helps regulate body weight trajectories in children and adolescents by increasing total energy expenditure, improving insulin sensitivity, and modulating lipid metabolism and inflammatory status. Regular physical activity has also been shown to support healthier body composition and metabolic profiles, thereby reducing the risk of overweight and obesity (19). Outdoor activity is widely recognized as a key protective factor against myopia. The high-intensity light environment outside is thought to stimulate retinal dopamine release, inhibiting excessive axial elongation and slowing myopia development. In addition, outdoor activity is typically accompanied by longer viewing distances and reduced near-work demands, which may alleviate accommodative stress and reduce hyperopic defocus, further contributing to the prevention of myopia (20).

Physical activity does not occur in isolation in daily life; rather, it occurs alongside sedentary behavior, screen exposure, and sleep patterns to constitute the overall daily activity structure of children and adolescents (21). Increased outdoor time and physical activity are often accompanied by reductions in near-work activities and sedentary time. This “time substitution effect” simultaneously reshapes the visual developmental environment and energy balance. Consequently, the influence of physical activity on myopia and obesity is likely to operate through multiple overlapping pathways, rather than through two independent mechanisms.

This mechanistic framework may also help explain why outdoor activity attenuates the association between overweight and incident myopia. Children with greater outdoor exposure are likely to experience a more favorable combination of light stimulation, reduced near-work burden, and increased movement-related energy expenditure, which may jointly weaken the behavioral coupling between excess weight and myopia risk.

On this basis, previous studies have proposed additional mechanistic clues linking anthropometric indicators with refractive development. On the one hand, metabolism-related factors may contribute to myopia development through nutritional and inflammatory pathways. Some evidence (22) suggests that higher saturated fat and cholesterol intake is associated with an increased risk of myopia in children, which is consistent with the possibility that dietary patterns and the metabolic states they reflect may indirectly influence refractive development through systemic inflammation, vascular function, or the retinal metabolic environment. On the other hand, the association between obesity and myopia may partly arise from shared behavioral foundations, particularly insufficient physical activity and reduced outdoor exposure (23, 24). A lack of physical activity, especially outdoor activity, may predispose children to both energy imbalance and reduced light exposure, while also coinciding with increased sedentary behavior and near-work time, thereby creating overlapping behavioral conditions relevant to both obesity and myopia.

These mechanistic considerations suggest that anthropometric indicators may be better understood as markers of broader metabolic status and lifestyle patterns rather than as direct causal agents in refractive development. However, these interpretations should remain cautious. Most of the included studies were observational and did not directly test mediation pathways, so the mechanisms discussed here should be understood as plausible and convergent explanatory hypotheses rather than demonstrated causal chains. Future longitudinal and intervention studies will be needed to clarify the relative contributions of metabolic, behavioral, and environmental pathways to the myopia–obesity relationship.

### 4.4 School environment as a strategic platform for co-prevention

This review demonstrates that only a small number of included studies have directly examined the school physical activity environment as a structural exposure in the joint prevention of myopia and obesity. Specifically, one national follow-up study provided the strongest direct evidence in this area, while one cluster randomized trial protocol and one randomized controlled intervention study positioned the school as the main implementation platform for integrated prevention. These studies generally operationalized the school physical activity environment using composite indicators that incorporated elements such as physical education scheduling, recess policies, the duration of school-based physical activity time, and the organization of sports activities, and then examined their associations with myopia-related outcomes, obesity-related outcomes, or comorbidity indicators. Here, upstream structural determinant refers to school-level contextual conditions, such as timetabling, physical education provision, recess policy, and access to outdoor space, that shape children's daily activity opportunities before individual behavior is expressed.

Although the number of such studies remains small, their findings are directionally consistent. The most direct evidence came from a limited number of school-focused studies, which showed not only separate associations between the school physical activity environment and myopia- and obesity-related outcomes, but also an independent association with the risk of myopia–obesity comorbidity. Importantly, these relationships remained significant even after adjustment for individual-level physical activity and family-related factors, suggesting that school environments may contribute above and beyond children's own behaviors or household characteristics. At the same time, this evidence base is still limited, and the current conclusions should therefore be interpreted as promising rather than definitive.

The strategic significance of schools in joint prevention is reflected in several ways. First, schools reach nearly all children and adolescents and therefore represent one of the most equitable and population-wide intervention platforms. Second, school timetables, recess arrangements, and campus environments shape children's daily opportunities for physical activity and outdoor exposure, creating “default exposures” that may be more sustainable than strategies relying solely on family initiative or individual motivation. Third, school-based physical activity initiatives can simultaneously address multiple behavioral risk factors, including sedentary behavior, excessive screen use, insufficient outdoor exposure, and psychosocial wellbeing, thereby offering the potential for broad, synergistic health benefits (25). However, the extent to which schools can fulfill this role is likely to vary across contexts, depending on differences in physical education provision, outdoor space, sports facilities, curriculum pressure, and implementation capacity. These contextual differences should be considered when interpreting the transferability of current findings across school systems.

### 4.5 A school-based co-prevention framework for myopia and obesity: conceptual structure and operational implications

Based on the synthesized evidence, we propose a school-based co-prevention framework to integrate the currently fragmented findings and to guide future research and practice related to myopia and obesity in children and adolescents. Rather than treating these two conditions as isolated outcomes, the framework situates them within a shared behavioral and environmental context in which physical activity and outdoor exposure occupy a central position.

This framework comprises four interconnected levels. The first is the upstream structural level, centered on the school physical activity environment, including physical education curricula, recess policies, campus design, and opportunities for physical activity and outdoor exposure. The second is the behavioral nexus level, in which physical activity and outdoor exposure function as core behavioral components within a broader daily activity system that also includes sedentary behavior, screen exposure, near-work activities, and sleep patterns. The third is the dual-outcome level, comprising myopia-related and obesity-related outcomes. The fourth is the interaction pathway level, which highlights potential bidirectional associations between weight status and visual development, as well as the possible moderating role of outdoor exposure in these relationships (Figure 2).

To make this framework more operational, the school physical activity environment in this review is defined as a set of school-level contextual and organizational conditions that shape children's routine opportunities for movement and outdoor exposure. In practical terms, this construct includes measurable components such as the frequency and duration of physical education classes, recess duration and activity structure, school-based outdoor time, access to playgrounds and sports facilities, and formal school policies related to outdoor exposure or organized sports. These components may be assessed as independent indicators or combined into composite school-environment measures, depending on study design and analytic purpose.

Within this framework, physical activity and outdoor exposure are operationalized as the key behavioral components positioned between school context and health outcomes. In turn, myopia-related outcomes may be represented by standardized refractive or vision indicators such as cycloplegic refraction, spherical equivalent refraction, axial length, or validated vision-screening outcomes, whereas obesity-related outcomes may be represented by BMI z-scores, overweight/obesity classification, waist circumference, body fat indicators, or waist-to-height ratio. This operational structure is intended to support more consistent measurement and improve comparability across future studies.

Figure 2 illustrates this framework visually. The school physical activity environment is positioned as an upstream structural context that may shape children's daily activity systems through physical education curricula, recess policies, outdoor spaces and facilities, and the organization of school-based activities. Physical activity and outdoor exposure are placed at the center of the framework as part of a broader behavioral nexus that also includes sedentary behavior, screen time, near-work, and sleep. From this perspective, visual and metabolic outcomes are not treated as entirely separate domains, but as related outcomes that may arise within overlapping behavioral and environmental systems.

In practical terms, this framework may be translated into school-based actions such as increasing the frequency and duration of physical education classes, protecting active recess time, incorporating structured outdoor activity into daily timetables, and improving access to outdoor spaces and sports facilities. Implementation may also benefit from coordination among schools, teachers, families, and local communities to support consistent movement-related and visual health behaviors across both school and home settings.

Future studies should test this framework using designs that are suitable for real-world school settings, including cluster randomized controlled trials, quasi-experimental natural experiments, and effectiveness–implementation hybrid designs. Evaluation may also benefit from implementation-science frameworks such as RE-AIM, which can assess not only intervention effectiveness, but also reach, adoption, implementation, and maintenance across school systems. Taken together, this framework is intended not only as a conceptual synthesis of the current evidence, but also as an operational scaffold for future school-based assessment, intervention design, and implementation-oriented research.

## 5 Conclusion

This review suggests that physical activity and outdoor exposure are the most actionable targets for the joint prevention of myopia and obesity in children and adolescents. From a practical perspective, schools may provide the most feasible setting for implementing integrated prevention strategies, because they can simultaneously shape opportunities for physical activity, outdoor exposure, and daily behavior patterns. However, the current evidence base remains largely observational, and stronger intervention evidence is still needed to support implementation.

At the research level, future studies should prioritize longitudinal cohort designs and integrated school-based intervention or implementation trials that simultaneously assess both myopia and obesity outcomes. Particular attention should be paid to key developmental stages, including preschool children, early school-age children, and adolescents, to clarify age-specific pathways and windows of prevention. Greater standardization is also needed in the measurement of physical activity, outdoor exposure, school physical activity environment, and myopia- and obesity-related outcomes to improve comparability across studies.

At the practice and policy level, schools and policymakers may consider strengthening physical education provision, protecting active recess time, incorporating structured outdoor activity into daily timetables, and improving access to outdoor spaces and sports facilities. Integrated school health policies may also benefit from stronger coordination among schools, teachers, families, and local communities. Overall, advancing this field will require not only stronger causal evidence, but also more implementation-oriented research to determine how integrated co-prevention strategies can be delivered effectively and sustainably in real-world educational systems.
