# Supplementary material for: Physical activity for the co-prevention of myopia and obesity in children and adolescents: a scoping review
Source: Front Public Health. 2026 May 18;14:1795516. doi: 10.3389/fpubh.2026.1795516 (PMC13223020; doi:10.3389/fpubh.2026.1795516)
Supplement: Supplementary file 1 [file Table_1.docx]

Supplementary Material

**Supplemental Table 1.** Database search blocks and final database-specific search strings.

| Database | Construct | Search blocks and final search string |
| --- | --- | --- |
| PubMed | Population | (child* OR adolescent* OR youth* OR student*) |
| PubMed | Physical activity | ("physical activity" OR exercise OR sport* OR "outdoor activity" OR "outdoor time" OR "time outdoors" OR "physical education" OR "school-based physical activity") |
| PubMed | Myopia | (myopia OR nearsighted* OR "refractive error" OR "axial length" OR "visual acuity") |
| PubMed | Obesity | (obesity* OR overweight OR "body mass index" OR BMI OR adiposity) |
| PubMed | Final search string | (("Child"[Mesh] OR "Adolescent"[Mesh] OR child*[Title/Abstract] OR adolescent*[Title/Abstract] OR youth*[Title/Abstract] OR student*[Title/Abstract]))  AND (("Motor Activity"[Mesh] OR "Exercise"[Mesh] OR "Sports"[Mesh] OR "physical activity"[Title/Abstract] OR exercise[Title/Abstract] OR sport*[Title/Abstract] OR "outdoor activity"[Title/Abstract] OR "outdoor time"[Title/Abstract] OR "time outdoors"[Title/Abstract] OR "physical education"[Title/Abstract] OR "school-based physical activity"[Title/Abstract]))  AND (("Myopia"[Mesh] OR myopia[Title/Abstract] OR nearsighted*[Title/Abstract] OR "refractive error"[Title/Abstract] OR "axial length"[Title/Abstract] OR "visual acuity"[Title/Abstract]))  AND (("Obesity"[Mesh] OR "Overweight"[Mesh] OR obesity*[Title/Abstract] OR overweight[Title/Abstract] OR "body mass index"[Title/Abstract] OR BMI[Title/Abstract] OR adiposity[Title/Abstract])) |
| Web of Science | Population | TS=(child* OR adolescent* OR youth* OR student*) |
| Web of Science | Physical activity | TS=("physical activity" OR "outdoor activity" OR "time outdoors" OR "physical education" OR "school-based physical activity" OR recess) |
| Web of Science | Myopia | TS=(myopia OR nearsighted* OR "refractive error"OR "axial length" OR "visual acuity") |
| Web of Science | Obesity | TS=(obesity* OR overweight OR "body mass index" OR BMI OR adiposity) |
| Web of Science | Final search string | TS=((child* OR adolescent* OR youth* OR student*) AND ("physical activity" OR exercise OR sport* OR "outdoor activity" OR "outdoor time" OR "time outdoors" OR "physical education" OR "school-based physical activity" OR recess) AND (myopia OR nearsighted* OR "refractive error" OR "axial length" OR "visual acuity") AND (obesity* OR overweight OR "body mass index" OR BMI OR adiposity)) |
| CNKI | Population | SU=(儿童 OR 青少年 OR 学生 OR child OR adolescent OR youth OR student) |
| CNKI | Physical activity | SU=(身体活动 OR 体育锻炼 OR 运动 OR 户外活动 OR 户外时间 OR 体育课 OR 学校身体活动环境 OR "physical activity" OR exercise OR sport OR "outdoor activity") |
| CNKI | Myopia | SU=(近视 OR 屈光不正 OR 眼轴 OR 视力 OR myopia OR "refractive error" OR "axial length" OR "visual acuity") |
| CNKI | Obesity | SU=(肥胖 OR 超重 OR 体重指数 OR BMI OR 脂肪 OR obesity OR overweight OR "body mass index") |
| CNKI | Final search string | SU=(儿童 OR 青少年 OR 学生 OR child OR adolescent OR youth OR student) AND SU=(身体活动 OR 体育锻炼 OR 运动 OR 户外活动 OR 户外时间 OR 体育课 OR 学校身体活动环境 OR "physical activity" OR exercise OR sport OR "outdoor activity") AND SU=(近视 OR 屈光不正 OR 眼轴 OR 视力 OR myopia OR "refractive error" OR "axial length" OR "visual acuity") AND SU=(肥胖 OR 超重 OR 体重指数 OR BMI OR 脂肪 OR obesity OR overweight OR "body mass index") |

**Note**: All databases were searched from inception to January 1, 2026, and the final search was executed on January 1, 2026. PubMed, Web of Science Core Collection, and CNKI were searched using database-specific syntax. Final search strings were constructed by combining the population, physical activity/outdoor exposure, myopia, and obesity blocks with Boolean operators. Language restrictions were limited to English and Chinese, and publication type was restricted to original research. In CNKI, SU= denotes the subject field.

**Supplemental Table 2.** PRISMA-ScR checklist and corresponding locations in the revised manuscript.

| Section | Item | PRISMA-ScR checklist item | Reported on page(s) |
| --- | --- | --- | --- |
| Title | | | |
| Title | 1 | Identify the report as a scoping review | 1 |
| Abstract | | | |
| Structured summary | 2 | Provide a structured summary including background, objectives, eligibility criteria, sources of evidence, methods, results, and conclusions | 1 |
| Introduction | | | |
| Rationale | 3 | Describe the rationale for the review and why a scoping review approach is appropriate | 1-3 |
| Objectives | 4 | Provide explicit questions and objectives (population, concept, context) | 3-5 |
| Methods | | | |
| Protocol and registration | 5 | Indicate whether a protocol exists and provide registration information | 4 |
| Eligibility criteria | 6 | Specify inclusion/exclusion criteria and rationale | 5-6 |
| Information sources | 7 | Describe all data sources and search dates | 6-7 |
| Search | 8 | Present full search strategy for at least one database | 6 |
| Selection of sources of evidence | 9 | Describe screening and selection process | 6-7 |
| Data charting process | 10 | Describe data extraction/charting methods | 6-7 |
| Data items | 11 | List and define variables extracted | 7 |
| Critical appraisal | 12 | Describe critical appraisal (if conducted) | 6 |
| Synthesis of results | 13 | Describe how data were handled and summarized | 3-4,7 |
| Results | | | |
| Selection of sources of evidence | 14 | Report number screened, included, excluded (with flow diagram) | 7 |
| Characteristics of sources | 15 | Present characteristics of included studies | 7-8 |
| Critical appraisal within sources | 16 | Present appraisal results (if done) | N/A |
| Results of individual sources | 17 | Present key findings from included studies | 8-10 |
| Synthesis of results | 18 | Summarize findings in relation to objectives | 8-10 |
| Discussion | | | |
| Summary of evidence | 19 | Summarize main findings and implications | 10-15 |
| Limitations | 20 | Discuss limitations | 10-11 |
| Conclusion | 21 | Provide interpretation and implications | 15-16 |
| Funding | | | |
| Funding | 22 | Describe funding sources and roles | 16 |

**Supplementary Table 3.** Summary of measurement approaches across included studies.

| Author (year) | Myopia indicator | Obesity indicator | Outdoor indicator |
| --- | --- | --- | --- |
| Yang et al. (2024) | Myopia: Eye examinations were performed by trained staff. Refractive error was measured using an autorefractor (Topcon KR-800) under cycloplegia (0.5% proparacaine + 1% cyclopentolate), and axial length was measured with the IOL Master V5.2. Myopia was defined as spherical equivalent refraction < -0.50 D. | Overweight/obesity: Height and weight were measured and BMI (kg/m^2^) was calculated; weight status was classified according to the Chinese screening standard for school-age children and adolescents. | Outdoor time: Objectively measured using a wearable smartwatch with UV/light sensor, GPS, and pedometer; total outdoor minutes were standardized to average minutes per day. |
| Liu S. et al. (2025) | Poor vision rather than myopia: Uncorrected visual acuity was assessed using a standard logarithmic visual acuity chart at 5 m; each eye was tested separately, and the worse eye was used if unequal; UCVA < 5.0 was defined as poor vision. | Overweight/obesity: Height and weight were measured with standardized equipment and BMI was calculated; overweight/obesity was classified by WS/T 586–2018. | Outdoor activity time: Collected by questionnaire and analyzed as a correlating factor; the paper confirms it was a questionnaire variable, but the exact item wording, unit, or grouping was not fully described in the main text. |
| Dang et al. (2024) | Myopia: Assessed using unaided distance visual acuity at 5 m with a logarithmic chart, together with non-cycloplegic refraction measured by a desktop automated computerized optometer. | Overweight/obesity: Height and weight were measured by trained technicians; BMI was calculated and categorized as overweight, obesity, and severe obesity using age- and sex-specific cutoffs. | Not individual outdoor time, but school PA environment: Assessed by a paper questionnaire covering 8 school physical activity environment indicators, including PA duration at school and outdoor PA duration; each favorable item scored 1 point, and the total score was categorized as unfavorable/moderate/favorable. |
| Drews-Botsch C & Harrington S (2025) | Vision problems rather than myopia: Defined by primary caregiver report of whether the child currently had, or had ever had, a vision problem requiring correction at ages 3 and/or 5; current vision problems were asked again at age 9. This was not an objective refraction-based myopia test. | Overweight/obesity: Height and weight were measured by study staff and BMI was calculated; BMI categories were based on WHO age- and sex-specific percentiles: excess weight (>85th percentile) and obesity (>95th percentile). | No separate outdoor indicator; instead, physical activity and sedentary behavior were measured. At age 9, children completed a time-use diary reporting minutes spent in physical play/exercise/sports and sedentary activities such as TV, leisure reading, computer use, and video games. |
| Yin et al. (2024) | Myopia: Determined by questionnaire-reported myopia/diopter information; because right and left eye spherical equivalents were highly correlated, only the right eye was used. Myopia was defined as right-eye refraction < -0.50 D, and further classified as mild and moderate-to-high myopia. | Weight status/obesity: Height and weight were measured uniformly and BMI was calculated according to Chinese standards; body fat percentage (BFP) was additionally measured using bioelectrical impedance analysis (InBody770). | No separate outdoor indicator; a physical activity questionnaire recorded weekly moderate-to-vigorous physical activity (MVPA), screen time, and average daily sleep duration. |
| Zong et al. (2024) | No direct myopia assessment; myopia was mentioned only as a background health concern. | Overweight/obesity: BMI was used as the weight-status indicator, and BMI was analyzed as non-overweight vs overweight in binary logistic regression. | Outdoor activity time: Collected via the parent-reported Family Questionnaire on Children’s Multimedia Usage. The key item was children’s “daily family life allocation,” using an 8-point scale (1 = 0 min, 8 = ≥2 h) to estimate outdoor activity and screen time. Physical health was additionally assessed with the CHeHCI/eHCI health dimension. |
| Zhang (2023) | Myopia: Assessed using a 5-m standard logarithmic visual acuity chart plus desktop automated computerized refraction. Myopia was defined as uncorrected visual acuity <5.0 and non-cycloplegic spherical equivalent < -0.50 D; myopia in either eye counted as myopia. | Obesity: Height and weight were measured by trained staff; BMI (kg/m²) was calculated and obesity was classified according to WS/T 586–2018. | No separate outdoor measure; lifestyle was assessed by questionnaire and summarized using the American Heart Association healthy lifestyle score across smoking, alcohol use, diet, exercise, screen time, and sleep duration. |
| Dong (2024) | Screening myopia: Derived from the student common disease monitoring system, including left and right visual acuity measurements; the paper focused on screening myopia combined with overweight/obesity. | Overweight/obesity: Also obtained from the student common disease monitoring form, based on height and weight screening. | Outdoor activity time: Measured by questionnaire using the past 1-week daytime outdoor activity time per day. The questionnaires also recorded average daily screen time over the past week and number of days with at least 60 min of MVPA; outdoor time was commonly analyzed as <2 h/day vs ≥2 h/day. |
| Zheng et al. (2024) | Myopia: Participants underwent a comprehensive ophthalmic examination. Refraction in both eyes was measured with an automatic refractive instrument (RK-F1, Canon) without cycloplegia, and axial length was measured using the IOLMaster. The right eye was used for analysis, and myopia was defined as SER ≤ -0.50 D. | Obesity: Height and weight were measured in a standardized manner, and BMI was categorized according to the WHO Asia-Pacific classification: Q1 (<25), Q2 (25.0–29.9), and Q3 (≥30.0 kg/m²). | Outdoor time: Students completed a lifestyle questionnaire with their parents. Outdoor time was defined as the total time spent in outdoor leisure and sports activities, calculated as [(weekday outdoor hours × 5) + (weekend outdoor hours × 2)] / 7. |
| Wang et al. (2023) | Study protocol, not a completed outcome paper. The primary outcomes include changes in visual acuity; the protocol states that distance vision will be tested separately in both eyes, and diopter will be measured with a desktop automated computerized optometrist, averaged three times per eye. | Outdoor/activity indicator: A self-designed questionnaire recorded time spent in different types of activities and school/family health-promotion behaviors; reported indicators included school-day outdoor activity time and weekend outdoor activity time. | Outdoor indicator: Primary behavioral outcomes include outdoor activity indicators. The intervention core is “22510SS”, in which the first “2” means 2 h of daytime outdoor activity. Outdoor activity, screen time, diet, sleep, and supervision are mainly assessed by questionnaires. |
| Yang (2025) | Screening myopia/vision: Assessed by unaided distance visual acuity testing plus non-cycloplegic computerized refraction. Screening myopia was defined as vision <5.0 in either eye plus spherical equivalent < -0.50 D, or current orthokeratology use; the worse eye was used for analysis. | Obesity/body composition: Routine examinations included height, weight, waist circumference, hip circumference, and blood pressure; bioelectrical impedance analysis was used to measure body fat percentage, fat mass, water percentage, muscle mass, and fat-free mass; BMI was calculated. | Outdoor/activity indicator: A self-designed questionnaire recorded time spent in different types of activities and school/family health-promotion behaviors; reported indicators included school-day outdoor activity time and weekend outdoor activity time. |
| Zhang (2025) | Not an original measurement study, but an outcome-indicator development study. Under the “effectiveness” dimension, the selected myopia-related outcomes were myopia incidence and spherical equivalent. | The obesity-related outcomes included BMI, prevalence of overweight/obesity, and waist-to-height ratio. | No separate outdoor test method was specified; behavior-level outcomes were represented by a comprehensive health behavior score within the RE-AIM implementation framework. |
| Drury et al. (2024) | No direct myopia assessment; myopia was discussed only as background. This was a qualitative focus-group study, not an objective ophthalmic study. | No direct obesity assessment; obesity was also addressed only as a background problem. | Outdoor/activity measure: The study used 4 focus groups (31 participants over 6 months) to explore barriers and facilitators to participation in a community-based outdoor intervention. Thus, it assessed feasibility and perceptions of outdoor participation, rather than a standardized quantitative outdoor exposure measure. |
